# Associations between local COVID-19 policies and anxiety in the USA: a longitudinal digital cohort study

**DOI:** 10.1136/bmjph-2024-001135

**Published:** 2025-01-16

**Authors:** Aaron E Cozen, Rita Hamad, Soo Park, Gregory M Marcus, Jeffrey E Olgin, Madelaine Faulkner Modrow, Amy Chiang, Matthew Brandner, Jaime H Orozco, Kristen Azar, Sylvia E K Sudat, Carmen R Isasi, Natasha Williams, Pelin Ozluk, Heather Kitzman, Sara J Knight, Ana Sanchez-Birkhead, John Kornak, Thomas Carton, Mark Pletcher

**Affiliations:** 1 Dept of Epidemiology and Biostatistics, University of California San Francisco, San Francisco, California, USA; 2 Department of Social & Behavioral Sciences, Harvard School of Public Health, Boston, Massachusetts, USA; 3 Division of Cardiology, University of California San Francisco, San Francisco, California, USA; 4 Division of General Internal Medicine, University of California San Francisco, San Francisco, California, USA; 5 Center for Health Systems Research, Sutter Health, Walnut Creek, California, USA; 6 Dept of Epidemiology and Population Health, Albert Einstein College of Medicine, Bronx, New York, USA; 7 Dept of Population Health, New York University Grossman School of Medicine, New York, New York, USA; 8 Elevance Health Inc, Indianapolis, Indiana, USA; 9 University of Texas Southwestern Medical Center, Dallas, Texas, USA; 10 Division of Epidemiology, University of Utah, VA Salt Lake City Healthcare System, Salt Lake City, Utah, USA; 11 College of Nursing, University of Utah, Salt Lake City, Utah, USA; 12 Louisiana Public Health Institute, New Orleans, Louisiana, USA

**Keywords:** COVID-19, Anxiety, Mental Health, Public Health, Sociodemographic Factors

## Abstract

**ABSTRACT:**

**Introduction:**

A lack of coordinated federal guidance led to substantial heterogeneity in local COVID-19 policies across US states and counties. Local government policies may have contributed to increases in anxiety and mental health disparities during the COVID-19 pandemic.

**Methods:**

We analysed associations between composite policy scores for containment and closure, public health or economic support from the US COVID-19 County Policy Database and self-reported anxiety scores (Generalised Anxiety Disorder-7) from COVID-19 Citizen Science participants between 22 April 2020 and 31 December 2021.

**Results:**

In 188 976 surveys from 36 711 participants in 100 counties across 28 states, associations between anxiety and containment and closure policy differed by employment (p<0.0001), with elevated anxiety under maximal policy for people working in hospitality and food services (+1.05 vs no policy; 95% CI: 0.45, 1.64) or arts and entertainment (+0.56; 95% CI 0.15, 0.97) and lower anxiety for people working in healthcare (−0.43; 95% CI −0.66 to –0.20) after adjusting for calendar time, county-specific effects and COVID-19 case rates and death rates. For public health policy, associations differed by race and ethnicity (p=0.0016), with elevated anxiety under maximal policy among participants identifying as non-Hispanic Black (+1.71; 95% CI 0.26, 3.16) or non-Hispanic Asian (+0.74; 95% CI 0.05, 1.43) and lower anxiety among Hispanic participants (−0.63, 95% CI −1.26 to –0.006). Associations with public health policy also differed by gender (p<0.0001), with higher anxiety scores under maximal policy for male participants (+0.42, 95% CI 0.09, 0.75) and lower anxiety for female participants (−0.40, 95% CI −0.67 to –0.13). There were no significant differential associations between economic support policy and sociodemographic subgroups.

**Conclusions:**

Associations between local COVID-19 policies and anxiety varied substantially by sociodemographic characteristics. More comprehensive containment policies were associated with elevated anxiety among people working in strongly affected sectors, and more comprehensive public health policies were associated with elevated anxiety among people vulnerable to racial discrimination.

**WHAT IS ALREADY KNOWN ON THIS TOPIC**

- COVID-19 policies varied widely across states and counties in the USA and may have contributed to increases in anxiety and other mental health problems during the pandemic.

**WHAT THIS STUDY ADDS**

- This study of over 36 000 participants from 100 US counties shows that some local COVID-19 policies were associated with increased anxiety among groups that disproportionately experienced adverse economic impacts, health effects or racial discrimination during the pandemic.

**HOW THIS STUDY MIGHT AFFECT RESEARCH, PRACTICE OR POLICY**

- Pandemic policies should consider potential effects on mental health, with special attention to economic and social risk factors in vulnerable populations.

## Introduction

 COVID-19 increased the prevalence of mental health disorders including clinically significant anxiety in the USA and many other countries.^1^,^3^ In the USA, the impacts of COVID-19 have been severe and highly unequal despite perceived advantages in pandemic readiness, exacerbating socioeconomic, racial/ethnic and gender disparities in mental health.^12 4^,^7^

Local policies varied substantially over the course of the pandemic and across different locations within the USA, where public health authority is highly decentralised and where many federal aid programmes were administered at the state or local level.^8^,^12^ Local policies may have alleviated or exacerbated worry about the health effects of COVID-19, economic precarity and other stressors associated with anxiety during the pandemic, many of which disproportionately affected vulnerable groups.^5 7 13^

We focused this investigation on three major domains of local policies identified in the US COVID-19 County Policy Database^8^ (UCCP): (1) containment and closure policies—including school and business closures, cancellation of public events, closures of public transport, stay-at-home requirements, restrictions on gatherings and curfews; (2) public health policies—including information campaigns, diagnostic testing programmes, vaccination programmes, masking requirements and access restrictions for indoor spaces; and (3) economic support policies—including supports for basic levels of income, housing, utilities and nutrition. Containment and mitigation policies may reduce anxiety by reducing perceived risk of infection or may increase anxiety by threatening employment or reducing healthy social interaction.^14 15^ Public health policies may decrease anxiety by reducing perceived health risks^16^ or may increase anxiety for people distressed about testing, vaccination or masking requirements. Containment policies and public health policies might also increase anxiety by increasing perceptions that the pandemic posed serious health risks. Anxiety and depression are strongly associated with economic precarity,^1 7 13 14^ and policies alleviating economic insecurity may improve mental health in economically vulnerable groups.^517^,^19^ The effects of different policies on anxiety may depend on demographic and social circumstances, specific measures and information campaigns used for policy implementation, changes in policy over time, trust in government policymakers, legislation affecting the authority of policymaking agencies and other individual and community characteristics.^20 21^

The heterogeneity in local COVID-19 policies over the course of the pandemic in the USA^8 9 22^ presents an opportunity to investigate their effects on anxiety and other outcomes. We linked policy data from the UCCP Database^8^ with survey responses from the COVID-19 Citizen Science Study^23^ (CCS) to investigate associations between the comprehensiveness of COVID-19-related policies in place at the county level and self-reported generalised anxiety during the first 20 months of the COVID-19 pandemic. We hypothesised that more comprehensive containment and closure policies would increase anxiety, especially for people working in strongly affected sectors; that public health policy would decrease anxiety among people at greater risk of COVID-19 infection or more vulnerable to severe COVID-19 disease; and that economic support policies would reduce anxiety among people vulnerable to economic insecurity.

## Methods

### Study design and sample

The CCS study is a longitudinal digital cohort study of adults 18 and over launched 26 March 2020 to study COVID-19 testing, outcomes and patient-reported impact of the pandemic.^23^ The study was approved by the University of California, San Francisco, Institutional Review Board (17–21879).

CCS participants were enrolled on a continuous, rolling basis, completing baseline surveys once at the time of enrollment and other recurring surveys about experiences and attitudes on a weekly or monthly basis. All surveys were administered in English, and participants recorded responses using a smartphone app or a web portal.^23^ CCS participants were included in our analysis if they completed at least 1 monthly anxiety survey between 22 April 2020 and 31 December 2021, along with baseline surveys about demographic characteristics including MacArthur-scale Subjective Social Status (SSS)^24^ and provided a US zip code that was linkable with a county for which policy data from the UCCP Database was available.

### Patient and public involvement

Participants were involved in the design and conduct of the CCS study via participant-proposed survey questions and iterative survey revisions in response to participant feedback, new study findings disseminated via the Eureka app and public study websites, and new research questions arising in the course of the research. Participants were involved in recruitment via word of mouth, news and social media coverage and outreach from partner studies and organisations.^23 25^

### Survey measurements

Our primary outcome was anxiety, measured in monthly surveys using the seven-item Generalised Anxiety Disorder (GAD-7) questionnaire. Responses to each item in the GAD-7 questionnaire are scored as integer values between 0 and 3 according to the frequency of symptoms during the preceding 2 weeks (‘Not at all’, ‘Several days’, ‘More than half the days’ or ‘Nearly every day’) and summed to a combined score between 0–21, where scores of ≥10 are considered indicative of moderate to severe anxiety disorders.^26 27^

Measurements of demographic characteristics from baseline CCS surveys used in analyses included SSS values between 1 (lowest) and 10 (highest),^24^ age, gender, race and ethnicity and type of employment. Participants selected one or more race categories from the following: ‘Black or African American’, ‘White’, ‘Asian (including South Asian and Asian Indian)’, ‘Native Hawaiian or Pacific Islander’, ‘American Indian or Alaska Native’, ‘Some other race’ and ‘Don’t know’. These responses were combined with self-reported Hispanic ethnicity to define five mutually exclusive categories of race and ethnicity; participants who selected more than one race were categorised as ‘Hispanic any race’ or ‘Non-Hispanic other’. Participant gender selections were similarly condensed from the following categories: ‘Female’, ‘Male’, ‘Genderqueer’, ‘Transgender Man’, ‘Transgender Woman’, ‘Another Gender Identity’ and ‘Not stated’. Missing CCS survey responses for covariates other than SSS and age were tabulated as ‘not stated’.

Participants’ primary residence zip codes at baseline were used to link to county policy scores from the UCCP, as well as corresponding county-level COVID-19 case and death rates from The New York Times.^8 28^ In each case, participant zip codes were linked to data for the county containing the largest proportion of zip code residences based on crosswalk files from the Department of Housing and Urban Development,^29^ and corresponding anxiety surveys were then linked by date to time-varying county policy data and COVID-19 rate data. County population statistics and metropolitan/non-metro categories were obtained from the US Department of Agriculture Economic Research Service.^30^

### Policy exposures

Records of COVID-19 policies for counties corresponding to participants’ primary residence zip codes were obtained from the US UCCP Database. Weekly data on 26 policy indicators were gathered from sources including government websites, press releases, news articles, response summaries, and databases and social media posts by government organisations.^8^ Policy data were available from 27 January 2020 to 31 December 2021 for 100 counties.^8^

The UCCP categorised individual policies into three overarching domains: containment and closure (13 policy indicators), public health measures (eight policy indicators) and economic support (five policy indicators).^8^ Containment and closure policies included mandated closures of schools and businesses (eg, restaurants), event cancellations, closure of public transportation, stay at home requirements, curfews and restrictions on gatherings; public health policies included information campaigns, masking requirements, diagnostic testing programmes, vaccination programmes and access restrictions for indoor spaces; economic support policies included income, housing, nutritional or utility support (online supplemental file 1). Specific policies within each domain were scored on ordinal, integer scales (eg, 0–3 or 0–4) according to comprehensiveness. Public health policies related to vaccination by group or vaccination by location were exceptions that were scored on a numeric scale (0–1) according to proportional availability (online supplemental file 1). Policies were imputed from county-level or city-level evidence when this indicated a specific policy in place, regardless of the activity level of the policy (including evidence for policy without any active measures). Policies were imputed from state-level evidence when there was no evidence for a county-level or city-level policy or when these policies specified deferral to state-level policy (online supplemental Tables S1, S2, S3). Policies were imputed from city-level evidence when this indicated administration at the city-level, and participants also reported a residence zip code within these cities. Evidence was tabulated as county-level for cities that comprised 100% of the corresponding county population (San Francisco, New York, Philadelphia and New Orleans). Policies were scored as zero where no county-level, state-level or city-level information was available to determine the policy in place or where policies prohibited any restrictive measures (eg, bans on policies requiring face coverings). Individual policy indicator scores were normalised to a 0–1 scale to weigh each equally before summing to provide a composite policy score for each policy domain (online supplemental file 1).

For descriptive analyses, we categorised counties as having relatively high (>median) or low (≤median) comprehensiveness of policies related to containment and closure, public health or economic support using their average composite policy score between 20 April 2020 and 31 December 2021. A list of counties included in our sample, their average scores in each policy domain, and the number of CCS participants and participant responses from each county are provided in online supplemental table S1.

We estimated recent exposure to time-varying local policies for each participant at the time of each anxiety survey response by averaging composite policy scores for each participant’s county during the preceding 4 weeks for primary analyses, and the preceding 2 weeks or 8 weeks for sensitivity analyses. To compare associations between anxiety and the comprehensiveness of policies for each composite policy domain, we normalised these rolling averages to a 0–1 scale, with 0 indicating a total absence of policy in that policy domain and 1 indicating the maximum possible composite policy score during that time frame.

### Statistical analysis

Analyses were performed using Stata v17 or R Statistical Software v4.3.2. Participants living in counties with low- vs high-average composite policy scores were described according to baseline demographics and other characteristics, and frequencies were compared using X^2^ tests.

To analyse associations between each of the three composite policy indices and anxiety, we implemented a linear mixed effects model regressing the GAD-7 anxiety score on all three composite scores of policy comprehensiveness over the preceding 4 week period. We adjusted for baseline characteristics, including SSS, age, gender, race and ethnicity and accounted for repeated measures from individual participants by including a random intercept for each participant. Age and SSS were included in models with linear and quadratic terms. We also adjusted for county-specific effects, for time-varying county-level COVID-19 case and death rates during the week of the anxiety response and for calendar time itself to account for temporal trends in anxiety during the study period, modelled as a 9 knot cubic spline of days elapsed following declaration of COVID-19 as a global pandemic by the WHO on 11 March 2020.

In a pre-planned sensitivity analysis, we analysed adjusted associations between policy scores and anxiety using 2 week or 8 week averages instead of 4 week averages to define the relevant policy exposure preceding an anxiety survey response.

To test whether associations between policies and anxiety differed for different population subgroups, we then fit a series of more complex models with the addition of two-way interaction terms between each of the three composite policy exposure scores over the preceding 4 week period and one of five participant characteristics—SSS, age, gender, race/ethnicity or type of employment—modelled as categorical variables. We interpreted interactions based on statistical significance (heterogeneity p values), and the magnitude of the subgroup-specific point estimates.

### Data availability

A version of the full data set used for all analyses is provided in online supplemental file 3. To protect participant privacy county FIPS codes are supplied in place of zip codes, age groups are supplied rather than specific ages in years, and observations from counties with <10 participants are omitted (removing 16 participants and 86 observations). The resulting data set includes 188 890 observations from 36 695 participants in 97 counties and 26 US states. Sources of evidence for all policy indices, counties and weeks are provided in online supplemental file 4.

## Results

52 351 CCS participants who reported SSS in baseline surveys and US zip codes that could be linked to COVID-19 case and death rate data completed 278 414 monthly anxiety surveys between 22 April 2020 and 31 December 2021 when UCCP policy data were also available. 36 711 (70%) of these participants reported zip codes corresponding to UCCP counties and completed 188 976 (68% of the total) anxiety surveys during this period (median 3 monthly anxiety surveys per participant, IQR 2–7). Included participants were linked to 100 counties and 28 states in the evolving UCCP database, with substantial regional and intraregional variation in the comprehensiveness of local policies over the course of the pandemic (online supplemental table S1, figure S1). Policies in place for these counties related to containment and closure or public health were imputed primarily from county-level evidence (65% and 60% of total policy-weeks analysed, respectively), whereas economic support policies were imputed from county-level and state-level evidence in roughly equal proportions (online supplemental table S2). Evidence for a local policy in place was missing most often for public health policies (24% of policy-weeks vs 7% of policy-weeks for containment and closure and 14% for economic support), for calendar periods early in the pandemic, and for counties in the Midwest and South (online supplemental Tables S2, S3 and S4). Composite policy scores in each of the three domains were not strongly correlated for counties and dates represented in anxiety surveys (R^2^=0.1 for public health vs containment and closure policy scores; R^2^=0.08 for economic support vs containment and closure policy scores; R^2^=0.05 for economic support vs public health policy scores).

The median age of participants in our analytic sample was 54 years. Participants were 66% female, 80% non-Hispanic White and 8% Hispanic, with 58% living in Western Census region states and 35% living in counties of the San Francisco Bay Area (San Francisco, Alameda, Santa Clara, San Mateo, Contra Costa, Marin, Solano, Napa, Sonoma). 21% of participants reported living with school-aged children (grades k-12). 87% of participants were from large metropolitan counties with populations of one million or more (72 of the 100 counties analysed), and 10% were from counties with populations of 250 000 to one million (22 counties; Online supplemental table S5). Participants living in counties with less active policies for containment and closure, public health or economic support were in the minority (34%, 39% and 37% of the sample, respectively) and were more predominantly White and non-Hispanic (table 1, online supplemental Table S6 and S7).

**Table 1:** Characteristics of COVID-19 citizen science participants living in counties with low vs high average containment and closure policy scores

Characteristic	Low containment and closure policy activity* (1.67–4.57) n=12 584	High containment and closure policy activity* (> 4.57–8.37) n=24 127	p value†
Age group (years)			
18–34	1639 (13.0%)	2899 (12.0%)	p<0.001
35–49	3716 (29.5%)	6740 (27.9%)	
50–64	3758 (29.9%)	7536 (31.2%)	
≥65	3471 (27.6%)	6952 (28.8%)	
Gender			
Female	8327 (66.2%)	15 808 (65.5%)	p=0.258
Male	4093 (32.5%)	7965 (33.0%)	
Other or not stated	164 (1.3%)	354 (1.5%)	
Race/ethnicity			
Non-Hispanic White	10 860 (86.3%)	18 335 (76.0%)	p<0.001
Hispanic, any race	765 (6.1%)	2091 (8.7%)	
Non-Hispanic Asian	280 (2.2%)	1979 (8.2%)	
Non-Hispanic Black	282 (2.2%)	498 (2.1%)	
Non-Hispanic Other	397 (3.2%)	1224 (5.1%)	
Subjective social status‡			
1–2	89 (0.7%)	187 (0.8%)	p<0.001
3–4	926 (7.4%)	1468 (6.1%)	
5–6	3471 (27.6%)	5547 (23.0%)	
7–8	6548 (52.0%)	12 736 (52.8%)	
9–10	1550 (12.3%)	4189 (17.4%)	
Employment			
Healthcare	2323 (18.5%)	4024 (16.7%)	p<0.001
Education	1517 (12.1%)	2762 (11.4%)	
Scientific and technical services	1068 (8.5%)	2684 (11.1%)	
Finance and insurance	756 (6.0%)	1222 (5.1%)	
Arts, entertainment and recreation	293 (2.3%)	961 (4.0%)	
Retail	270 (2.1%)	381 (1.6%)	
Manufacturing	325 (2.6%)	298 (1.2%)	
Hospitality and food services	170 (1.4%)	384 (1.6%)	
Other or not stated	5862 (46.6%)	11 411 (47.3%)	

* Counties were stratified according to average weekly policy comprehensiveness below or above the median across counties for containment and closure (4.57) for the period between 22 April 2020 and 31 December 2021. See Methods.

† X2 test.

‡ MacArthur-scale Subjective Social Status values between 1 and 10, combined into five groups.

**Figure 1:**
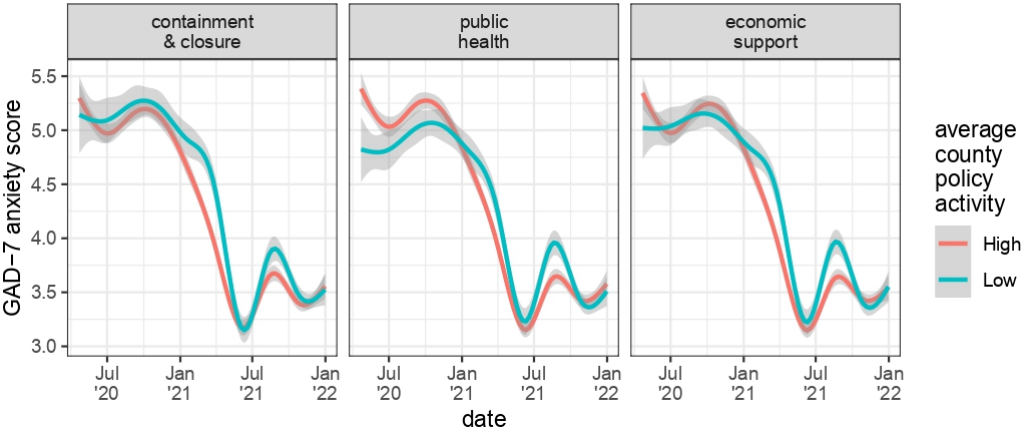
Generalised Anxiety Disorder-7 anxiety scores by local policy comprehensiveness. Lines show smoothed conditional means of anxiety over time, stratified by the average policy comprehensiveness in each of three overarching policy domains for participants’ counties of residence between 22 April 2020 and 31 December 2021 (see Methods). Shaded gray areas show 95% CIs.

GAD-7 anxiety survey scores were highest during the early months of the pandemic (figure 1). On average, anxiety scores were similar across US census regions, with slightly higher scores for participants in the Northeast, and during calendar periods when COVID-19 case and death rates were highest (table 2). Unadjusted time trends in anxiety were similar for counties with generally less comprehensive policies vs those with more comprehensive policies over the course of the study period for the three domains we examined (figure 1).

**Table 2:** Policy exposure and anxiety survey scores (Generalised Anxiety Disorder-7) by calendar date, region and COVID-19 case and death rates

Characteristic	Responses (n=1 88 976)	Mean (SD)			
		Containment and closure policy index in the last 4 weeks*	Public health policy index in the last 4 weeks*	Economic support policy index in the last 4 weeks*	GAD-7 anxiety score†
Calendar date range					
2020-04-22 - 2020-12-05	47 376	8.1 (1.7)	4.2 (0.8)	4.4 (0.7)	5.1 (4.9)
2020-12-06 - 2021-05-25	47 348	6.7 (2.1)	5.5 (0.8)	4.3 (0.8)	4.2 (4.6)
2021-05-26 - 2021-10-07	47 207	1.7 (1.6)	5.8 (0.8)	4.2 (0.9)	3.5 (4.3)
2021-10-08 - 2021-12-31	47 045	1.0 (1.4)	5.6 (0.9)	3.7 (1.0)	3.4 (4.3)
US census region					
West	125 107	5.1 (3.6)	5.4 (1.0)	4.5 (0.6)	4.1 (4.5)
Midwest	28 428	3.2 (2.7)	5.1 (1.0)	3.4 (1.0)	3.9 (4.6)
South	19 953	3.3 (3.0)	4.3 (0.9)	3.1 (0.8)	3.8 (4.4)
Northeast	15 488	2.6 (3.1)	5.2 (0.9)	4.0 (0.7)	4.5 (4.9)
Average COVID-19 cases/100 k in participants' county‡					
≤5	38 191	6.0 (3.1)	5.2 (1.3)	4.5 (0.7)	3.9 (4.5)
>5–10	42 751	4.7 (3.3)	5.3 (1.0)	4.3 (0.8)	3.9 (4.5)
>10–20	50 147	3.7 (3.5)	5.3 (1.0)	4.1 (0.9)	4.1 (4.6)
>20	57 887	3.8 (3.6)	5.2 (0.9)	3.9 (1.0)	4.2 (4.6)
Average COVID-19 deaths/100 k in participants' county‡					
≤0.07	53 106	4.2 (3.5)	5.4 (1.1)	4.4 (0.7)	3.9 (4.5)
>0.08–0.14	44 922	4.1 (3.5)	5.2 (1.1)	4.2 (0.9)	4.0 (4.5)
>0.15–0.26	44 342	3.8 (3.3)	5.4 (1.0)	4.0 (0.9)	4.0 (4.6)
>0.27	46 606	5.5 (3.5)	5.0 (1.0)	4.0 (1.0)	4.3 (4.7)

* Rolling 4 week averages of local policy comprehensiveness scores, using a normalised scale in which each containment and closure, public health or economic support policy subcategory was scored from 0 to 1.

† Generalised Anxiety Disorder (GAD-7) score on a scale of 0 to 21.

‡ Rolling weekly averages of county-level COVID-19 cases or deaths per 100 000 from the New York Times.

GAD-7, Generalised Anxiety Disorder-7.

Composite policy scores in each domain were not associated with significant differences in anxiety scores in the overall study population after adjusting for participant characteristics, random participant-level effects, calendar time, COVID-19 case and death rates, county-specific effects and other confounders (table 3). By contrast, sociodemographic characteristics including SSS, age, gender, race/ethnicity and field of employment were each associated with significant differences in anxiety scores (online supplemental table S8). Sensitivity analysis using either 2 week or 8 week rolling averages of policy comprehensiveness rather than the 4 week rolling averages showed similar results, with a trend towards lower anxiety associated with more comprehensive policies for public health or economic support using 8 week rolling averages (online supplemental table S9).

**Table 3:** Associations between COVID-19 policies and self-reported anxiety

Policy*	Adjusted increase in GAD-7 anxiety score associated with each policy index (95%CIs)†	
	Estimate	P value
**Composite policy indices**		
Containment and closure	−0.03 (-0.20, 0.15)	0.77
Public health	−0.13 (-0.38, 0.12)	0.31
Economic support	−0.12 (-0.30, 0.05)	0.17

* Policy exposures calculated by averaging the mean policy comprehensiveness score in the participant’s county of residence for the 4 week period prior to the anxiety survey response. Models were constructed using composite policy indices in each of the three policy domains (summary scores for containment and closure, economic support and public health) scaled to a score between 0 and 1 to reflect the comprehensiveness of local policy. See Methods.

† Generalised Anxiety Disorder (GAD)-7 surveys were used to measure anxiety, scored from 0 to 21. Coefficients presented represent the estimated increase in the GAD-7 score associated with maximal exposure to the given policy vs no exposure for 4 weeks prior to the survey response. All models were adjusted to account for random participant-level effects (using a random intercept for each participant), sociodemographic characteristics as fixed time-invariant factors, county-specific effects, calendar time (spline) and time-varying weekly COVID-19 case and death rates. See Methods.

**Figure 2:**
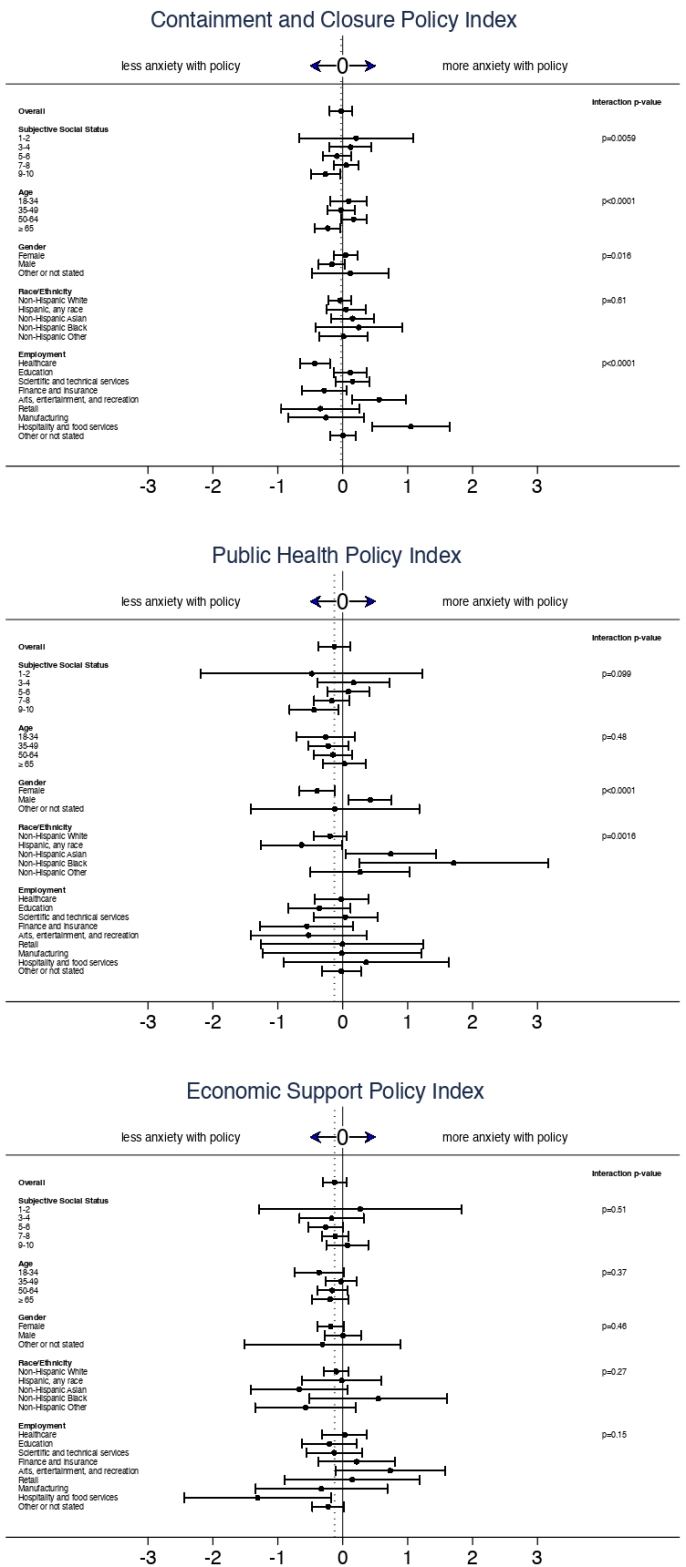
Model estimates for differential associations between policy comprehensiveness and anxiety related to participant characteristics. Panels show estimated differences in Generalised Anxiety Disorder-7 anxiety scores under maximal local policy in each respective policy domain. Dotted vertical lines show estimates for the overall study population after adjusting for participant demographic characteristics, COVID-19 case and death rates, and calendar time. Points show adjusted differential associations by participant characteristics. Whiskers show 95% CIs. Omnibus p values are shown for each interaction model.

Interaction analyses showed that associations between policy comprehensiveness and anxiety varied significantly by participant characteristics, including field of employment, race/ethnicity, gender identity and SSS (figure 2, online supplemental table S10). With maximal levels of containment and closure policy, anxiety scores were elevated for people employed in fields related to hospitality and food services (+1.05 vs no policy; 95% CI 0.45, 1.64) or arts, entertainment and recreation (+0.56; 95% CI 0.15, 0.97), and anxiety scores were lower for people working in healthcare (−0.43; 95% CI −0.66 to –0.20). Associations for more comprehensive containment and closure policy also differed by SSS and age group, with lower anxiety scores for participants in the highest quintile of SSS (−0.27; 95% CI −0.49 to –0.04) and for participants who were at least 65 years old at the time of enrollment (−0.23; 95% CI −0.43 to –0.03).

Associations for public health policy differed by race and ethnicity, with elevated anxiety scores under more comprehensive policy for people self-identifying as non-Hispanic Black (+1.71; 95% CI 0.26, 3.16) or non-Hispanic Asian (+0.74; 95% CI 0.05, 1.43) and lower anxiety for people who identified as Hispanic (−0.63; 95% CI −1.26 to –0.006). Associations for public health policy also differed by gender, with higher anxiety for male participants (+0.42; 95% CI 0.09, 0.75) and lower anxiety for female participants (−0.40; 95% CI −0.67 to –0.13) under a more comprehensive policy. Associations with economic policy did not differ significantly by any of the participant characteristics analysed.

## Discussion

Our analysis of nearly 190 000 anxiety survey responses from over 36 000 participants in 100 US counties across 28 states between April 2020 and December 2021 shows high levels of anxiety early in the pandemic that declined over time. The comprehensiveness of local policy related to containment and closure, public health or economic support during the 4 week period preceding anxiety surveys was not associated with significant differences in anxiety scores in the overall study population. However, associations between containment and closure policy and public health policy varied substantially by participant characteristics, especially type of employment and race/ethnicity.

We found that relationships between anxiety and containment and closure policy differed by field of employment, with notably elevated anxiety among persons working in arts and entertainment or in hospitality and food services. These increases in anxiety may have been driven by severe economic impacts of containment and closure among affected workers in these sectors,^31^,^35^ outweighing perceived reductions in infection risk due to containment policies. By contrast, a more comprehensive containment and closure policy was associated with slightly lower anxiety among healthcare workers, who were highly impacted by surges in COVID-19 cases, but less affected by employment insecurity.

More comprehensive public health policy was associated with higher anxiety among Black participants and Asian participants and lower anxiety among Hispanic participants. Public health measures such as diagnostic testing and vaccination notably required in-person interactions that might increase exposure to racial bias or anxiety about racial discrimination for people from marginalised racial and ethnic groups.^36 37^ Anxiety levels of Black participants in particular were strikingly elevated under more comprehensive public health policy—this association was the largest in magnitude among all subgroup interactions, though the CI was wide. Higher anxiety among Black participants under more comprehensive public health policy may reflect distrust in vaccination programmes, diagnostic testing and other public health measures related to a long and continuing history of discrimination by government and medical institutions, racialised politics during the 2020 US presidential election, and highly publicised racialised violence before and during the pandemic.^638^,^42^ Elevated anxiety among Asian participants may reflect politically motivated racialisation of SARS-CoV-2 by government officials and public figures and intensification of anti-Asian racism and violence during the pandemic.^6 43 44^ Similar to Black people, Hispanic people in the USA experienced pronounced disparities in mortality among working-age adults and associated with in-person employment in ‘essential’ occupational sectors, as well as racial discrimination associated with delaying or foregoing medical care.^3745^,^47^ In contrast to Black participants, a more comprehensive public health policy was associated with a moderate decrease in anxiety among Hispanic participants, suggesting contrasting perceptions about diagnostic testing programmes, vaccination campaigns, mask requirements or other public health policies.

Although younger age was significantly associated with higher anxiety in this study, similar to other studies,^2 3^ and older age is among the best-established risk factors for the severity of illness with SARS-CoV-2 infection, associations between local policy and anxiety did not depend strongly on age in our analyses. Under maximal containment and closure policies, anxiety levels were slightly lower for people 65 and over. Associations between anxiety and the comprehensiveness of public health policy or economic policy did not differ significantly by age group.

Despite the profound effects of the pandemic on employment and economic insecurity, and evidence that loss of employment and economic precarity are strongly associated with anxiety and other adverse metal health outcomes,^1 7 13 14 35 48 49^ we did not find any significant associations between composite measures of economic policy and anxiety. There is evidence that state policies supporting Medicaid benefits, unemployment insurance, suspended utility shutoffs and prohibitions on evictions and foreclosures during the pandemic may have helped ameliorate anxiety related to economic insecurity.^5 17^ Our composite index for economic support incorporated scores for some of these measures, as well as others. The lack of evidence for associations between economic support policy and anxiety in our analyses could indicate that programmes supporting nutrition, housing, utilities and other basic needs primarily benefited highly disadvantaged groups that were not well represented in this study cohort. Alternatively, it is possible that our use of composite policy scores, our focus on policies during a relatively short period preceding anxiety surveys, or other aspects of our analysis obscured associations related to specific economic support policies, including differential associations among specific sociodemographic groups.

Previous studies have reported relatively modest or nonsignificant associations between the stringency of government COVID-19 containment and closure policies and anxiety in non-probability cohorts in the UK and Canada.^50 51^ These results are consistent with the lack of associations in our overall study population and underscore the importance of analyses to investigate differential associations in vulnerable and minoritised sociodemographic subgroups that are often underrepresented in volunteer and convenience samples. While our analyses identified associations between anxiety and local policy comprehensiveness that differed significantly by sociodemographic characteristics, many of these are modest in magnitude compared with more pronounced associations between anxiety and health concerns or economic stress identified in previous analyses of the COVID-19 Citizen Science Study cohort and other studies^7 13 15^ and compared with differences in anxiety over the course of the pandemic associated directly with socioeconomic status, gender, age and other characteristics.^1^,^36 13 52^ The most conspicuous association in our analyses is the highly elevated anxiety levels among Black participants under more comprehensive public health policies, which merits special attention in light of the disproportionate morbidity and mortality suffered by Black Americans during the pandemic, compounding longstanding disparities in health and life expectancy, and the already high cumulative economic burden of poor health among Black people in the USA in years preceding the pandemic.^45^,^4753 54^ The strongly elevated anxiety levels among people working in hospitality and food services under more comprehensive containment and closure policies are also notable, highlighting the competing stresses of occupational exposure to risk of infection and job insecurity for people working in these sectors.^34^ Potential approaches for mitigation include sustained investment in infrastructure supporting accessible primary care and public health communication within marginalised and low-income communities and expansion of economic assistance and health insurance for people suffering from income insecurity and loss of employment.^1842 55^,^57^

Strengths of our analysis include a large and diverse participant cohort that encompassed many US counties and states, a longitudinal design spanning acute early phases of the pandemic as local policies were developed and implemented, and the use of systematically curated policy data from the US COVID-19 County Policy Database.^8^ Our analysis also had important limitations. First, the CIs and p values reported by our models should be interpreted cautiously given that these were based on Gaussian distributions, whereas the GAD-7 scores in our data set are zero-inflated (many zero scores). In addition, participants and counties analysed were not randomly selected. Our study sample was skewed towards participants who were female, non-Hispanic, White and highly educated, and it overrepresented the San Francisco Bay Area. As a result, our findings may not generalise to vulnerable sociodemographic groups and communities that are not well represented in this cohort, including people with limited English language proficiency, people with limited access to smartphones or computers and people living in rural areas. Subgroups that showed strong associations—including people who identified as Black or employed in service sector jobs—were each a relatively small fraction of the sample population. And our analyses based on composite policy indicators and generalised sociodemographic categories may mask important associations for specific or intersectional population subgroups, such as lower-income adults living with children, Hispanic agricultural workers or healthcare workers with lower occupational standing.^18 47 58 59^ These intersectional characteristics might differ in important ways within sociodemographic subgroups in a contrasting cohort, such as a sample more predominantly drawn from non-metropolitan counties, where temporal and sociodemographic patterns of COVID-19 waves also differed from more urbanised areas.^60 61^ Nevertheless, we successfully enrolled many participants with lower SSS, non-White race or Hispanic ethnicity from 100 UCCP counties across 28 US states, allowing us to adjust for key characteristics and conduct analyses with adequate power to detect contrasting associations in population subgroups.

Despite our efforts to adjust for individual characteristics, county-specific effects and time-varying factors associated with anxiety, residual confounding is an important possibility. While moderate clustering of policies within counties limits the power of correlational studies to resolve causal relationships,^8^ composite policy scores for each of the three policy domains were not strongly correlated for counties and dates analysed in this study. The UCCP Database may not include all relevant co-occurring governmental programmes and actions, and our approach combining policy activity into three composite policy scores may obscure the effects of specific policies, including differences related to the source of local policies and interactions at different levels of government involved in their implementation. These intergovernmental interactions notably included laws limiting or expanding state or local public health authority, with potential effects that warrant further investigation.^21 62^ Our scoring method also did not differentiate cases where no policy data was available vs cases where available evidence indicated that there was no active policy in place or cases where policies prohibited any restrictive measures (all were scored as zero). In addition, temporal relationships between policies and anxiety or other outcomes may differ by policy type and may be affected by the recency of any changes in policy and the implementation of these changes. Results of our sensitivity analyses show that more comprehensive policies for public health of economic support were each associated with lower anxiety in the overall study population when policy comprehensiveness was averaged over the preceding 8 week period but not when averaged over 2 week or 4 week periods. The effect of policy *changes* on anxiety and other outcomes might depend on the recency of the policy change, the comprehensiveness of the preceding policy in place, cumulative policy changes over the course of the pandemic and other factors, presenting complex analysis challenges that are outside the scope of this study. Finally, the effects of policies related to one set of activities, such as containment and closure, might also depend on policies in another area, such as economic support, as well as participant characteristics and experiences—interactions that might be complex, but important. These myriad interactions are beyond the scope of this analysis but are compelling for further investigation.

The pandemic exacerbated socioeconomic, racial and gender disparities associated with poor mental health in the USA.^1 2 4 13^ Prior analyses of state and local policies suggest mixed results addressing these impacts, where some policies may have been effective in protecting physical health, mental health and economic well-being, while others may have contributed to disparities in pandemic impacts related to socioeconomic inequalities in employment and community characteristics.^563^,^68^ Our analyses suggest that in aggregate, local policies related to containment and closure, public health or economic support may have had relatively limited immediate benefits in alleviating anxiety and that some local policies may have contributed to increased anxiety within vulnerable groups.

In conclusion, this study provides important evidence that local pandemic policies may have increased anxiety and mental health disparities in vulnerable groups. In the absence of strong federal guidance, local and state policymakers made decisions about which policies to implement, and how comprehensive those policies should be, often weighing potential benefits in controlling the spread of COVID-19 against potential harms, including economic, social and political consequences. Our findings suggest that differential effects of policies on mental health should be considered in future pandemic policies to buffer those with precarious employment, people marginalised by historic and ongoing racial discrimination and other social risk factors.

## Supplementary material

10.1136/bmjph-2024-001135 online supplemental file 1

10.1136/bmjph-2024-001135 online supplemental file 2

10.1136/bmjph-2024-001135 online supplemental file 3

10.1136/bmjph-2024-001135 online supplemental file 4

## Data Availability

All data relevant to the study are included in the article or uploaded as supplementary information.
