# Supplementary material for: Associations between local COVID-19 policies and anxiety in the USA: a longitudinal digital cohort study
Source: BMJ Public Health. 2025 Jan 16;3(1):e001135. doi: 10.1136/bmjph-2024-001135 (PMC11812870; doi:10.1136/bmjph-2024-001135)
Supplement: online supplemental file 1 [file bmjph-3-1-s001.pdf]

Policy Domain	Policy Variable	Description	Ordinal scoring	Normalized scoring	Additional Scoring Notes
Containment & Closure	irc1_school	closing of public K-12 schools (including independent school districts)	0 - No measures (i.e., no restrictions)<br>1 - Recommend closing or all schools open with alterations resulting in significant differences compared to non-Covid-19 operations<br>2 - Require closing only some levels or categories - e.g., just high school<br>3 - Require closing all levels<br>0 – No information available to determine policy in place	0 - No measures (i.e., no restrictions)<br>0.33 - Recommend closing or all schools open with alterations resulting in significant differences compared to non-Covid-19 operations<br>0.66 - Require closing only some levels or categories - e.g., just high school<br>1 - Require closing all levels<br>0 – No information available to determine policy in place	
Containment & Closure	irc2_workplace	closings of workplaces (limited to non-essential office work).	0 - No measures (i.e., no restrictions)<br>1 - Recommend closing (or recommend work from home)<br>2 - Require closing (or work from home) for some sectors or categories of non-essential office workers<br>3 - Require closing (or work from home) all-but-essential workplaces (e.g., grocery stores, doctors, pharmacies)<br>0 – No information available to determine policy in place	0 - No measures (i.e., no restrictions)<br>0.33 - Recommend closing (or recommend work from home)<br>0.66 - Require closing (or work from home) for some sectors or categories of non-essential office workers<br>1 - Require closing (or work from home) all-but-essential workplaces (e.g., grocery stores, doctors, pharmacies)<br>0 – No information available to determine policy in place	
Containment & Closure	irc3_events	cancelling public events	0 - No measures (i.e., no restrictions)<br>1 – Events allowed, with minimal (>=50% capacity) limitations<br>2 – Events allowed, with major (<50% capacity) limitations<br>3 - Recommend cancelling<br>4 - Require cancelling<br>0 – No information available to determine policy in place	0 - No measures (i.e., no restrictions)<br>0.25 – Events allowed, with minimal (>=50% capacity) limitations<br>0.5 – Events allowed, with major (<50% capacity) limitations<br>0.75 - Recommend cancelling<br>1 - Require cancelling<br>0 – No information available to determine policy in place	
Containment & Closure	irc4_private_gather	restrictions on private gatherings	0 - No measures (i.e., no restrictions)<br>1 - Restrictions on very large gatherings (the limit is above 1000 people)<br>2 - Restrictions on gatherings between 101-1000 people<br>3 - Restrictions on gatherings between 11-100 people<br>4 - Restrictions on gatherings of 10 people or less<br>0 – No information available to determine policy in place	0 - No measures (i.e., no restrictions)<br>0.25 - Restrictions on very large gatherings (the limit is above 1000 people)<br>0.5 - Restrictions on gatherings between 101-1000 people<br>0.75 - Restrictions on gatherings between 11-100 people<br>1 - Restrictions on gatherings of 10 people or less<br>0 – No information available to determine policy in place	
Containment & Closure	irc5_public_transpo	restrictions on public transport	0 - No measures (i.e., no restrictions)<br>1 - Recommend closing (or significantly reduce volume/route/means of transport available)<br>2 - Require closing (or prohibit most from using it)<br>0 – No information available to determine policy in place	0 - No measures (i.e., no restrictions)<br>0.5 - Recommend closing (or significantly reduce volume/route/means of transport available)<br>1 - Require closing (or prohibit most from using it)<br>0 – No information available to determine policy in place	
Containment & Closure	irc6_stay_home	stay at home requirements	0 - No measures (i.e., no restrictions)<br>1 - Recommend not leaving house<br>2 - Require not leaving house with exceptions for daily exercise, grocery shopping, and 'essential' trips<br>3 - Require not leaving house with minimal exceptions (e.g., allowed to leave only once a week, or only one person can leave at a time)<br>0 – No information available to determine policy in place	"0 - No measures (i.e., no restrictions)<br>0.33 - Recommend not leaving house<br>0.66 - Require not leaving house with exceptions for daily exercise, grocery shopping, and 'essential' trips<br>1 - Require not leaving house with minimal exceptions (e.g., allowed to leave only once a week, or only one person can leave at a time)<br>0 – No information available to determine policy in place"	
Containment & Closure	irc9_gym	restrictions on gyms	0 - No measures (i.e., no restrictions)<br>1 – Open, with minimal (>=50% capacity) limitations<br>2 – Open, with major (<50% capacity) limitations<br>3 – Closed<br>0 – No information available to determine policy in place	0 - No measures (i.e., no restrictions)<br>0.33 – Open, with minimal (>=50% capacity) limitations<br>0.66 – Open, with major (<50% capacity) limitations<br>1 – Closed<br>0 – No information available to determine policy in place	

Policy Domain	Policy Variable	Description	Ordinal scoring	Normalized scoring	Additional Scoring Notes
Containment & Closure	irc10_restaurant	restrictions on restaurants	0 - No measures (i.e., no restrictions)<br>1 – Open for indoor dining, with minimal (>=50% capacity) limitations<br>2 – Open for indoor dining, with major (<50% capacity) limitations<br>3 - Outdoor only (with or without takeout/delivery)<br>4 - Takeout/delivery only<br>5 – Closed<br>0 – No information available to determine policy in place	0 - No measures (i.e., no restrictions)<br>0.2 – Open for indoor dining, with minimal (>=50% capacity) limitations<br>0.4 – Open for indoor dining, with major (<50% capacity) limitations<br>0.6 - Outdoor only (with or without takeout/delivery)<br>0.8 - Takeout/delivery only<br>1 – Closed<br>0 – No information available to determine policy in place	
Containment & Closure	irc11_bar	restrictions on bars	0 - No measures (i.e., no restrictions)<br>1 – Open for indoor drinking, with minimal (>=50% capacity) limitations<br>2 – Open for indoor drinking, with major (<50% capacity) limitations<br>3 - Outdoor only (with or without takeout/delivery)<br>4 - Takeout/delivery only<br>5 – Closed<br>0 – No information available to determine policy in place	0 - No measures (i.e., no restrictions)<br>0.2 – Open for indoor drinking, with minimal (>=50% capacity) limitations<br>0.4 – Open for indoor drinking, with major (<50% capacity) limitations<br>0.6 - Outdoor only (with or without takeout/delivery)<br>0.8 - Takeout/delivery only<br>1 – Closed<br>0 – No information available to determine policy in place	
Containment & Closure	irc12_movie	restrictions on movie theaters	0 - No measures (i.e., no restrictions)<br>1 – Open, with minimal (>=50% capacity) limitations<br>2 – Open, with major (<50% capacity) limitations<br>3 – Closed<br>0 – No information available to determine policy in place	0 - No measures (i.e., no restrictions)<br>0.33 – Open, with minimal (>=50% capacity) limitations<br>0.66 – Open, with major (<50% capacity) limitations<br>1 – Closed<br>0 – No information available to determine policy in place	
Containment & Closure	irc13_daycare	restrictions on day care centers	0 - No measures (i.e., no restrictions)<br>1 - Recommend closing or all day cares open with alterations resulting in significant differences compared to non-Covid-19 operations<br>2 - Require closing (only some levels or categories - e.g., babies)<br>3 - Require closing all levels<br>0 – No information available to determine policy in place	0 - No measures (i.e., no restrictions)<br>0.33 - Recommend closing or all day cares open with alterations resulting in significant differences compared to non-Covid-19 operations<br>0.66 - Require closing (only some levels or categories - e.g., babies)<br>1 - Require closing all levels<br>0 – No information available to determine policy in place	
Containment & Closure	irc15_religious_gathering	restrictions on religious gatherings	0 - No measures (i.e., no restrictions)<br>1 – Open, with minimal (>=50% capacity) limitations<br>2 – Open, with major (<50% capacity) limitations<br>3 – Closed<br>0 – No information available to determine policy in place	0 - No measures (i.e., no restrictions)<br>0.33 – Open, with minimal (>=50% capacity) limitations<br>0.66 – Open, with major (<50% capacity) limitations<br>1 – Closed<br>0 – No information available to determine policy in place	
Containment & Closure	irc17_curfew	curfew requirements	0 - No measures (i.e., no restrictions)<br>1 – Required<br>0 – No information available to determine policy in place	0 - No measures (i.e., no restrictions)<br>1 – Required<br>0 – No information available to determine policy in place	
Public Health	irh1_campaigns	public information campaigns	0 - No COVID-19 public information campaign<br>1 - Public officials urging caution about COVID-19<br>2 - Coordinated public information campaign (e.g., across traditional and social media)	0 - No COVID-19 public information campaign<br>0.5 - Public officials urging caution about COVID-19<br>1 - Coordinated public information campaign (e.g., across traditional and social media)	
Public Health	irh2_testing	testing policy	0 - No testing policy<br>1 - Only those who both (a) have symptoms AND (b) meet specific criteria (e.g., essential workers, admitted to hospital, came into contact with a known case, returned from overseas)<br>2 - Testing of anyone showing COVID-19 symptoms<br>3 - Open public testing (e.g., “drive-through” testing available to asymptomatic people)<br>0 – No information available to determine policy in place	0 - No testing policy<br>0.33 - Only those who both (a) have symptoms AND (b) meet specific criteria (e.g., essential workers, admitted to hospital, came into contact with a known case, returned from overseas)<br>0.66 - Testing of anyone showing COVID-19 symptoms<br>1 - Open public testing (e.g., “drive-through” testing available to asymptomatic people)<br>0 – No information available to determine policy in place	
Public Health	irh3_contact_tracing	government contact tracing	0 - No contact tracing<br>1 - Limited contact tracing (not done for all cases)<br>2 - Comprehensive contact tracing (done for all identified cases)<br>0 – No information available to determine policy in place	0 - No contact tracing<br>0.5 - Limited contact tracing (not done for all cases)<br>1 - Comprehensive contact tracing (done for all identified cases)<br>0 – No information available to determine policy in place	

Policy Domain	Policy Variable	Description	Ordinal scoring	Normalized scoring	Additional Scoring Notes
Public Health	irh6a_covering_in	facial coverings in indoor settings	0 - No policy<br>88 – Ban on mask mandates (partial or total)<br>1 - Required in some specified indoor shared/public spaces outside the home<br>2 - Required in all indoor shared/public spaces outside the home<br>0 – No information available to determine policy in place	0 - No policy<br>0 – Ban on mask mandates (partial or total)<br>0.5 - Required in some specified indoor shared/public spaces outside the home<br>1 - Required in all indoor shared/public spaces outside the home<br>0 – No information available to determine policy in place	Bans on restrictive policy scored as 0
Public Health	irh6b_covering_out	facial coverings in outdoor settings	0 - No policy<br>88 – Ban on mask mandates (partial or total)<br>1 - Required in some specified outdoor shared/public spaces outside the home<br>2 - Required in all outdoor shared/public spaces outside the home<br>0 – No information available to determine policy in place	0 - No policy<br>0 – Ban on mask mandates (partial or total)<br>0.5 - Required in some specified outdoor shared/public spaces outside the home<br>1 - Required in all outdoor shared/public spaces outside the home<br>0 – No information available to determine policy in place	Bans on restrictive policy scored as 0
Public Health	irh7_vax_group	vaccine availability by group	N/A<br>Higher proportion of availability among the age group or "high-risk" group	0 - No vaccine availability in a specific state or county, as applicable, during the timeframe in which vaccines are available in other areas<br>0.X - Vaccine available to some groups<br>1 - Vaccine available to all age groups or "high-risk" groups, or universally available<br>0 – No information available to determine policy in place	**Proportion of vaccine availability by age group:**<br>☐ Age group 0-4 ☐ Age group 5-11 ☐ Age group 12-17<br>☐ Age group 18-29 ☐ Age group 30-39 ☐ Age group 40-49<br>☐ Age group 50-64 ☐ Age group 65-74 ☐ Age group 75-84<br>☐ Age group 85+<br><br>**Proportion of vaccine availability by "high risk" group:**<br>☐ Clinically vulnerable/shielding/chronic illness (those with significant underlying health conditions, not including the elderly) ☐ People with disabilities ☐ Residents of nursing homes/long-term care facilities<br>☐ Pregnant women ☐ People living with a vulnerable/ shielding person or other priority group (multigenerational fall within this)<br>☐ Racial/ethnic minorities ☐ Healthcare workers/carers<br>☐ Military (active and/or veteran) ☐ Police / First Responders<br>☐ Teachers or those working in educational settings ☐ Airport/border/airline staff ☐ Factory workers ☐ Other essential workers (e.g., utility, transportation, communications, critical manufacturing) ☐ Frontline retail workers (e.g., service industry, grocery, pharmacy, restaurants, nightclubs, retail stores)<br>☐ General workforce (available to anyone with a job) ☐ Students university/college ☐ Universal availability ☐ Other ________________________
Public Health	irh8_vax_org_loc	vaccine availability by organization/location	N/A<br>Proportion of availability across different locations	0 - No vaccine availability in a specific state or county, as applicable, during the timeframe in which vaccines are available in other areas<br>0.X - Vaccine available in some locations<br>1 - Vaccine available in all locations<br>0 – No information available to determine policy in place	**Proportion of vaccine availability by location**<br>0 - No availability<br>☐ Public health agencies<br>☐ Hospitals<br>☐ Clinics (including urgent care centers and dental clinics)<br>☐ Nursing homes/long-term care facilities<br>☐ Pharmacies<br>☐ Grocery stores<br>☐ Mass vaccination sites (e.g., sports arenas, unused retail space, malls, museums, recreation centers)<br>☐ Places of worship<br>☐ Schools (K-12)<br>☐ Colleges/universities<br>☐ Mobile vaccination units/community pop-up events<br>☐ Other (free text option) ____<br>0 – No information available to determine policy in place
Public Health	irh10_event_access	space/event access restrictions	0 – No policy<br>88 – Ban on policies restricting access<br>1 – Restricted access to indoor shared/public spaces based on proof of vaccination or recent negative Covid-19 test in only limited situations<br>2 – Restricted access to indoor shared/public spaces based on proof of vaccination or recent negative Covid-19 test in all situations (minor exceptions allowed)<br>0 – No information available to determine policy in place	0 – No policy<br>0 – Ban on policies restricting access<br>0.5 – Restricted access to indoor shared/public spaces based on proof of vaccination or recent negative Covid-19 test in only limited situations<br>1 – Restricted access to indoor shared/public spaces based on proof of vaccination or recent negative Covid-19 test in all situations (minor exceptions allowed)<br>0 – No information available to determine policy in place	Bans on restrictive policy scored as 0

Policy Domain	Policy Variable	Description	Ordinal scoring	Normalized scoring	Additional Scoring Notes
Economic Support	ire5_income	income support	0 - No measures (i.e., no support)<br>1 – Unemployment financial support only<br>2 – Other income support only<br>3 – Both unemployment financial support and other income support<br>0 – No information available to determine policy in place	0 - No measures (i.e., no support)<br>0.33 – Unemployment financial support only<br>0.66 – Other income support only<br>1 – Both unemployment financial support and other income support<br>0 – No information available to determine policy in place	
Economic Support	ire6_housing	housing support	0 - No measures (i.e., no support)<br>1 – Support<br>0 – No information available to determine policy in place	0 - No measures (i.e., no support)<br>1 – Support<br>0 – No information available to determine policy in place	
Economic Support	ire7_utility	utility support	0 - No measures (i.e., no support)<br>1 – Support<br>0 – No information available to determine policy in place	0 - No measures (i.e., no support)<br>1 – Support<br>0 – No information available to determine policy in place	
Economic Support	ire8_sickleave	paid sick leave	0 - No measures (i.e., no support)<br>1 – Support<br>0 – No information available to determine policy in place	0 - No measures (i.e., no support)<br>1 – Support<br>0 – No information available to determine policy in place	
Economic Support	ire9_nutrition	nutrition support	0 - No measures (i.e., no support)<br>1 – Support<br>0 – No information available to determine policy in place	0 - No measures (i.e., no support)<br>1 – Support<br>0 – No information available to determine policy in place	
		**Composite Variable**	**Composite Scoring Scale**		
		Containment and Closure policy comprehensiveness index	sum of normalized containment policy scores (0-13)		
		Public Health policy comprehensiveness index	sum of normalized Health policy scores (0-8)		
		Economic Support policy comprehensiveness index	sum of normalized economic policy scores (0-5)		
