# Supplementary material for: Associations between local COVID-19 policies and anxiety in the USA: a longitudinal digital cohort study
Source: BMJ Public Health. 2025 Jan 16;3(1):e001135. doi: 10.1136/bmjph-2024-001135 (PMC11812870; doi:10.1136/bmjph-2024-001135)
Supplement: online supplemental file 2 [file bmjph-3-1-s002.pdf]

Table S1. Policy indices and stratification by county.

County	State	Region[1]	participants	observations	High versus low policy average[2]			Policy Index Mean (SD)[3]		
					CC	PH	ES	CC	PH	ES
SAN FRANCISCO	CA	W	4,268	25,816	High	High	High	5.77 (3.78)	5.62 (0.94)	4.66 (0.00)
ALAMEDA	CA	W	2,417	15,738	High	High	High	6.11 (3.57)	5.64 (0.85)	4.97 (0.18)
SALT LAKE	UT	W	1,699	3,763	Low	Low	Low	4.20 (2.78)	3.82 (0.79)	3.20 (0.71)
SANTA CLARA	CA	W	1,477	8,634	High	High	High	5.66 (3.77)	5.01 (1.06)	4.42 (0.50)
COOK	IL	MW	1,415	7,047	Low	High	Low	4.19 (3.21)	5.20 (1.06)	3.43 (1.02)
SAN MATEO	CA	W	1,315	7,876	High	High	Low	5.36 (3.44)	5.50 (0.61)	3.66 (0.00)
CONTRA COSTA	CA	W	1,168	7,890	High	Low	High	5.29 (3.75)	4.71 (0.79)	4.66 (0.00)
NEW YORK	NY	NE	1,117	3,382	High	Low	High	5.18 (3.32)	4.37 (0.87)	4.66 (0.03)
MARIN	CA	W	1,031	6,603	High	High	High	5.62 (3.66)	5.43 (0.70)	4.66 (0.00)
DALLAS	TX	S	940	2,745	High	Low	Low	8.37 (0.88)	3.59 (0.66)	3.19 (0.22)
LOS ANGELES	CA	W	897	5,557	High	High	High	5.68 (3.73)	5.72 (1.01)	4.72 (0.13)
KINGS	NY	NE	847	2,509	High	High	High	5.15 (3.74)	4.90 (0.99)	4.46 (0.40)
MILWAUKEE	WI	MW	756	3,354	High	High	High	4.65 (2.81)	5.15 (0.87)	4.51 (0.31)
SONOMA	CA	W	712	4,653	High	High	High	5.48 (3.57)	5.78 (0.82)	4.91 (0.26)
NASSAU	NY	NE	678	1,833	Low	Low	High	4.56 (2.89)	4.33 (0.70)	4.25 (0.32)
SACRAMENTO	CA	W	611	3,525	High	High	High	5.88 (3.80)	5.89 (1.21)	4.53 (0.50)
KING	WA	W	591	4,322	High	High	High	5.20 (3.31)	4.86 (1.32)	4.22 (0.77)
COLLIN	TX	S	498	1,418	Low	Low	Low	1.67 (0.25)	3.26 (0.58)	3.14 (0.28)
TARRANT	TX	S	479	1,488	Low	Low	Low	3.35 (3.48)	3.70 (0.60)	2.82 (0.57)
QUEENS	NY	NE	465	1,293	High	High	Low	5.35 (2.91)	4.90 (0.99)	3.82 (0.91)
SAN DIEGO	CA	W	448	2,976	High	High	High	5.94 (3.84)	5.56 (0.68)	4.89 (0.35)
SANTA CRUZ	CA	W	441	2,358	High	High	High	6.16 (3.90)	5.56 (0.98)	5.00 (0.00)
BRONX	NY	NE	420	1,097	High	High	High	4.99 (3.62)	4.85 (0.95)	4.01 (0.53)
SUFFOLK	NY	NE	401	1,289	High	Low	High	4.59 (2.75)	4.27 (1.16)	4.33 (0.00)
WAUKESHA	WI	MW	386	1,737	Low	Low	Low	3.10 (1.90)	4.15 (0.72)	3.00 (0.58)
LAKE	IL	MW	377	1,827	Low	High	High	4.48 (3.18)	5.40 (0.65)	4.55 (0.74)
WILLIAMSON	TX	S	356	978	Low	Low	Low	2.84 (2.75)	4.49 (0.73)	3.16 (0.38)
WESTCHESTER	NY	NE	353	1,143	Low	Low	High	4.55 (3.20)	4.33 (1.32)	4.07 (0.34)
DAVIS	UT	W	326	774	Low	Low	Low	4.11 (2.79)	3.97 (0.63)	3.30 (0.59)
TRAVIS	TX	S	323	1,306	Low	Low	Low	3.04 (2.53)	3.18 (0.93)	3.22 (0.16)
DENTON	TX	S	312	1,030	Low	Low	Low	2.77 (2.65)	4.17 (0.80)	3.22 (0.16)
ORANGE	CA	W	304	2,035	High	High	High	5.64 (3.66)	5.50 (0.91)	4.96 (0.18)
DUPAGE	IL	MW	304	1,533	Low	Low	High	4.42 (2.41)	4.82 (0.75)	4.61 (0.47)
MARICOPA	AZ	W	297	1,836	Low	High	Low	3.45 (2.13)	4.85 (0.72)	3.88 (0.30)
ORLEANS	LA	S	293	1,228	Low	Low	Low	3.96 (2.54)	4.78 (0.81)	2.50 (0.92)
PLACER	CA	W	276	1,319	High	Low	High	6.16 (3.48)	4.82 (0.92)	5.00 (0.00)
HENNEPIN	MN	MW	264	1,842	High	Low	Low	4.72 (3.13)	3.76 (0.93)	3.19 (0.86)
KANE	IL	MW	256	1,141	Low	High	High	4.34 (3.01)	5.46 (0.78)	4.79 (0.39)
BELL	TX	S	249	557	Low	Low	Low	2.93 (2.54)	3.42 (0.94)	2.91 (0.84)
ST TAMMANY	LA	S	219	611	Low	Low	Low	3.56 (2.28)	3.44 (0.73)	3.09 (0.62)
MULTNOMAH	OR	W	216	1,629	High	Low	High	5.51 (3.73)	4.19 (0.94)	3.95 (0.73)
SOLANO	CA	W	208	1,441	High	High	High	6.07 (3.47)	5.11 (1.09)	4.48 (0.88)
MONTGOMERY	MD	S	198	1,512	Low	Low	Low	3.64 (2.77)	4.59 (0.88)	3.23 (0.68)
DENVER	CO	W	185	1,242	Low	High	High	4.36 (3.48)	5.13 (0.81)	4.31 (0.08)
RACINE	WI	MW	183	717	Low	Low	Low	4.14 (2.64)	4.32 (0.73)	3.60 (0.23)
DISTRICT OF COLUMBIA	DC	S	179	1,163	High	High	High	4.62 (3.26)	4.89 (0.79)	4.55 (0.28)
UTAH	UT	W	178	584	Low	Low	Low	3.31 (1.85)	4.27 (0.64)	3.30 (0.59)
OZAUKEE	WI	MW	173	710	Low	Low	Low	3.03 (1.10)	4.12 (0.72)	2.69 (0.48)
HUMBOLDT	CA	W	166	1,241	High	High	High	5.17 (3.42)	5.91 (0.95)	4.98 (0.15)
HARRIS	TX	S	164	1,027	Low	Low	Low	3.44 (3.09)	4.26 (0.64)	3.57 (0.29)
FAIRFAX	VA	S	159	1,149	Low	Low	Low	4.04 (2.99)	4.15 (0.99)	3.33 (0.00)
FRANKLIN	OH	MW	158	1,025	Low	High	Low	3.17 (2.57)	4.83 (0.67)	2.95 (0.68)
STANISLAUS	CA	W	158	921	High	Low	High	6.00 (3.47)	4.47 (1.26)	5.00 (0.00)
SAN JOAQUIN	CA	W	156	833	High	High	High	6.43 (3.49)	5.54 (1.08)	5.00 (0.00)
SHEBOYGAN	WI	MW	156	695	Low	Low	Low	4.10 (2.72)	4.18 (0.78)	2.62 (0.46)
BROWN	WI	MW	155	624	Low	Low	Low	3.85 (2.62)	3.77 (0.85)	2.81 (0.83)
JEFFERSON	LA	S	150	601	Low	High	High	4.11 (2.13)	5.16 (0.68)	4.04 (0.58)
KENOSHA	WI	MW	147	615	Low	Low	Low	4.18 (1.63)	4.37 (0.76)	2.73 (0.49)
RIVERSIDE	CA	W	146	1,055	High	High	High	5.80 (3.63)	5.30 (0.93)	4.69 (0.47)
FRESNO	CA	W	145	893	High	High	High	5.94 (3.76)	5.07 (1.11)	4.92 (0.27)

County	State	Region[1]	participants	observations	High versus low policy average[2]			Policy Index Mean (SD)[3]		
					CC	PH	ES	CC	PH	ES
NAPA	CA	W	136	1,020	High	High	High	5.62 (3.55)	5.17 (0.86)	5.00 (0.00)
MONTEREY	CA	W	135	863	High	High	High	6.24 (3.38)	5.28 (0.85)	4.70 (0.31)
CLARK	NV	W	134	754	High	Low	Low	4.57 (2.23)	4.10 (1.07)	3.06 (0.23)
DANE	WI	MW	128	898	Low	Low	Low	4.17 (2.79)	3.94 (0.60)	3.54 (0.33)
PHILADELPHIA	PA	NE	127	787	High	High	Low	5.20 (3.48)	5.08 (1.00)	3.79 (0.54)
BRAZOS	TX	S	121	298	Low	Low	Low	3.32 (1.76)	2.88 (0.68)	3.22 (0.16)
OAKLAND	MI	MW	120	791	Low	High	Low	4.23 (3.00)	5.24 (0.52)	2.51 (1.12)
ST LOUIS	MO	MW	113	656	High	Low	Low	5.82 (1.84)	4.72 (0.95)	2.20 (0.99)
PIMA	AZ	W	111	723	High	High	Low	5.22 (2.08)	5.37 (1.15)	2.90 (0.60)
RAMSEY	MN	MW	109	814	Low	Low	Low	4.52 (3.04)	4.43 (0.82)	3.66 (0.56)
ALLEGHENY	PA	NE	109	669	Low	Low	High	3.29 (2.58)	4.66 (0.45)	4.04 (0.61)
MONTGOMERY	PA	NE	104	747	Low	Low	High	3.44 (2.55)	4.59 (0.50)	4.04 (0.61)
EAST BATON ROUGE	LA	S	103	338	Low	Low	Low	3.54 (2.01)	3.84 (0.96)	3.31 (0.46)
VENTURA	CA	W	101	730	High	High	High	5.61 (4.09)	5.57 (1.06)	4.84 (0.43)
SNOHOMISH	WA	W	100	771	High	Low	High	5.25 (3.40)	4.62 (1.00)	4.29 (1.20)
BEXAR	TX	S	100	577	Low	Low	Low	4.04 (3.39)	4.61 (0.57)	3.22 (0.16)
WAYNE	MI	MW	98	591	Low	Low	Low	4.54 (3.17)	4.13 (0.51)	3.77 (0.60)
BOULDER	CO	W	95	665	Low	High	High	4.28 (3.55)	5.33 (0.72)	3.96 (0.21)
CUYAHOGA	OH	MW	93	546	Low	Low	Low	4.04 (2.63)	3.79 (0.78)	3.11 (0.78)
WASHTENAW	MI	MW	92	730	High	High	Low	4.95 (3.42)	5.12 (0.72)	3.84 (0.62)
EL DORADO	CA	W	92	523	High	High	High	5.56 (3.78)	4.91 (0.89)	5.00 (0.00)
SAN LUIS OBISPO	CA	W	91	610	High	High	High	5.08 (3.40)	5.38 (0.97)	4.66 (0.00)
ADA	ID	W	89	555	Low	Low	Low	3.04 (3.26)	4.18 (0.80)	2.45 (0.57)
NEVADA	CA	W	89	555	High	High	High	5.63 (3.63)	5.16 (0.85)	5.00 (0.00)
ARAPAHOE	CO	W	86	641	Low	High	High	4.40 (3.40)	4.86 (1.01)	4.17 (0.29)
JOHNSON	KS	MW	84	535	Low	Low	Low	3.84 (2.94)	4.68 (0.86)	2.37 (1.12)
HONOLULU	HI	W	80	520	High	Low	Low	7.25 (1.85)	3.94 (0.69)	3.17 (0.73)
DEKALB	GA	S	78	509	Low	High	Low	3.05 (2.25)	5.46 (1.43)	2.32 (0.41)
ARLINGTON	VA	S	76	556	Low	Low	Low	3.61 (2.35)	4.26 (1.17)	3.21 (0.33)
DAVIDSON	TN	S	70	438	Low	High	Low	4.39 (3.08)	5.03 (0.76)	3.20 (0.37)
CLACKAMAS	OR	W	61	428	High	Low	High	5.29 (3.48)	4.10 (0.82)	3.95 (0.73)
BALTIMORE	MD	S	53	378	Low	Low	High	3.44 (2.36)	3.98 (0.74)	4.01 (0.48)
MIDDLESEX	MA	NE	44	292	High	High	Low	4.88 (3.51)	5.52 (0.69)	2.87 (0.60)
SUFFOLK	MA	NE	38	193	High	High	High	5.52 (3.86)	5.29 (0.79)	4.35 (0.50)
ADAMS	CO	W	35	235	Low	High	High	4.49 (3.44)	5.08 (1.04)	3.93 (0.18)
WORCESTER	MA	NE	24	133	High	Low	Low	5.01 (3.59)	4.74 (0.80)	3.47 (0.94)
NORFOLK	MA	NE	10	81	High	High	Low	4.94 (3.45)	5.07 (0.42)	3.81 (0.94)
CHATHAM	NC	S	7	46	Low	High	Low	4.20 (3.06)	4.92 (0.90)	3.91 (0.36)
ESSEX	MA	NE	6	31	High	High	High	4.87 (3.57)	4.86 (0.74)	4.00 (0.51)
FAIRFIELD	CT	NE	3	9	High	High	High	5.04 (3.13)	5.58 (0.65)	4.84 (0.37)

[1] US Census regions: West (W), Midwest (MW), Northeast (NE), South (S)

[2] Counties were stratified according to average weekly policy comprehensiveness below or above the median across counties for containment and closure (CC; 4.57), public health (PH; 4.83), or economic support (ES; 3.92) for the period between April 22, 2020 – Dec 31, 2021. See Methods.

[3] A per-county mean and standard deviation of composite policy scores using a normalized scale in which each policy containment and closure (CC), public health (PH), or economic support (ES) policy subcategory was scored from 0-1.

Figure S1. Pandemic policy comprehensiveness for individual counties.

Lines show the comprehensiveness of policies related to containment and closure, public health, or economic support in individual counties from March 2, 2020 – December 31, 2021, with different color shades for states within each US Census region. Weekly policy scores within each domain were divided by maximum possible values to give measures of comprehensiveness normalized to a 0-1 scale. Black lines show smoothed conditional means for counties within each US Census region.

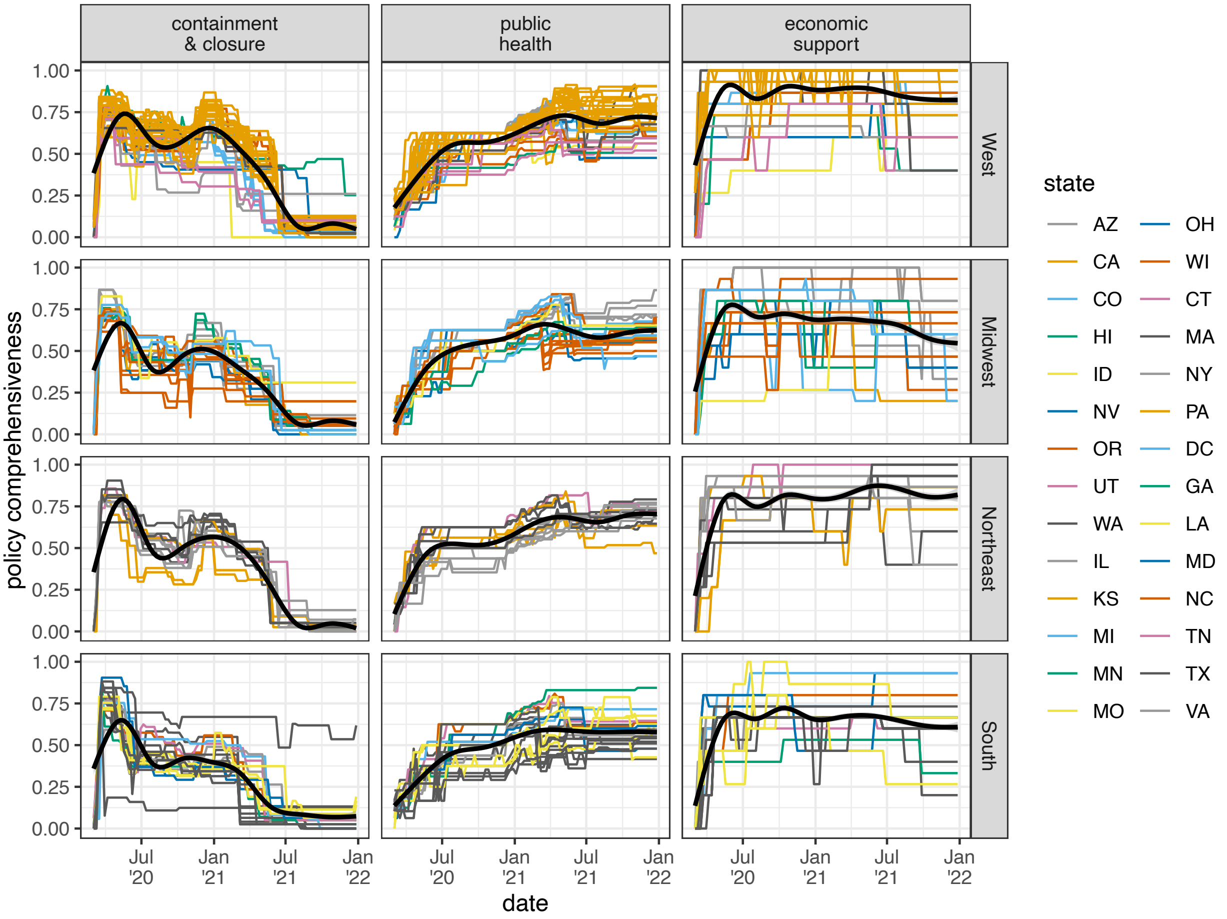

Table S2. Summary of evidence source for policies by policy domain[1].

Policy domain	County evidence	City evidence	State evidence	No evidence	total
Containment and Closure[2]	80733 (64.7%)	5893 (4.7%)	29223 (23.4%)	8951 (7.2%)	124800 (100%)
Public Health[3]	45980 (59.9%)	3534 (4.6%)	9220 (12%)	18066 (23.5%)	76800 (100%)
Economic Support[4]	20578 (42.9%)	2074 (4.3%)	18798 (39.2%)	6550 (13.6%)	48000 (100%)

[1] Policy-weeks are summarized as the product of (individual policies * weeks * counties) for 100 counties and 96 weeks between March 2, 2020, eight weeks before the first anxiety survey, and December 31, 2021.
[2] 13 individual containment and closure policies
[3] 8 individual public health policies
[4] 5 individual containment and closure policies

Table S3. Summary of evidence source for policies by calendar period[1].

Calendar period[2]	County evidence	City evidence	State evidence	No evidence	total
2020-03-02 - 2020-05-24	12506 (40.1%)	1136 (3.6%)	6868 (22%)	10690 (34.3%)	31200 (100%)
2020-05-25 - 2020-08-16	17167 (55%)	1272 (4.1%)	6899 (22.1%)	5862 (18.8%)	31200 (100%)
2020-08-17 - 2020-11-08	17877 (57.3%)	1353 (4.3%)	6449 (20.7%)	5521 (17.7%)	31200 (100%)
2020-11-09 - 2021-01-31	19288 (61.8%)	1453 (4.7%)	6867 (22%)	3592 (11.5%)	31200 (100%)
2021-02-01 - 2021-04-25	20195 (64.7%)	1621 (5.2%)	7085 (22.7%)	2299 (7.4%)	31200 (100%)
2021-04-26 - 2021-07-18	20169 (64.6%)	1641 (5.3%)	7485 (24%)	1905 (6.1%)	31200 (100%)
2021-07-19 - 2021-10-10	19931 (63.9%)	1512 (4.8%)	7898 (25.3%)	1859 (6%)	31200 (100%)
2021-10-11 - 2021-12-31	20158 (64.6%)	1513 (4.8%)	7690 (24.6%)	1839 (5.9%)	31200 (100%)

[1] Policy-weeks are summarized as the product of (individual policies * weeks * counties) for 26 individual policies and 100 counties.
[2] Eight 12-week periods

Table S4. Summary of evidence source for policies by US Census region[1].

US Census region	County evidence	City evidence	State evidence	No evidence	total
West	73725 (72%)	0 (0%)	16820 (16.4%)	11791 (11.5%)	102336 (100%)
Midwest	29169 (55.6%)	0 (0%)	15343 (29.3%)	7904 (15.1%)	52416 (100%)
Northeast	17365 (43.5%)	8627 (21.6%)	9415 (23.6%)	4529 (11.3%)	39936 (100%)
South	27032 (49.2%)	2874 (5.2%)	15663 (28.5%)	9343 (17%)	54912 (100%)

[1] Policy-weeks are summarized as the product of (individual policies * weeks * counties) for 26 individual policies and 96 weeks between March 2, 2020, eight weeks before the first anxiety survey, and December 31, 2021.

Table S5. Counties and participants by Rural-Urban Continuum Classification[1].

Rural-Urban Continuum Classification	counties	participants (%)
Metro - Counties in metro areas of 1 million population or more	72	32043 (87.3)
Metro - Counties in metro areas of 250,000 to 1 million population	22	3791 (10.3)
Metro - Counties in metro areas of fewer than 250,000 population	4	622 (1.7)
Nonmetro - Urban population of 20,000 or more, adjacent to a metro area	1	89 (0.2)
Nonmetro - Urban population of 20,000 or more, not adjacent to a metro area	1	166 (0.5)

[1] Rural-Urban Continuum classifications for individual counties from the USDA Economic Research Service. See Methods.

Table S6. Characteristics of COVID-19 Citizen Science participants living in counties with low versus high average public health policy scores.

Characteristic	Value	Low public health policy activity (2.88 - 4.83) n = 14,307	High public health policy activity (> 4.83 - 5.91) n = 22,404	p-value[2]
Age group (years)	18-34	1,663 (11.6%)	2,875 (12.8%)	p<0.001
	35-49	3,878 (27.1%)	6,578 (29.4%)	
	50-64	4,308 (30.1%)	6,986 (31.2%)	
	≥ 65	4,458 (31.2%)	5,965 (26.6%)	
Gender	Female	9,378 (65.5%)	14,757 (65.9%)	p=0.001
	Male	4,765 (33.3%)	7,293 (32.6%)	
	Other or not stated	164 (1.1%)	354 (1.6%)	
Race/ethnicity	Non-Hispanic White	12,174 (85.1%)	17,0231 (76.0%)	p<0.001
	Hispanic, any race	910 (6.4%)	1,946 (8.7%)	
	Non-Hispanic Asian	402 (2.8%)	1,857 (8.3%)	
	Non-Hispanic Black	310 (2.2%)	470 (2.1%)	
	Non-Hispanic Other	511 (3.6%)	1,110 (5.0%)	
Subjective social status[3]	1 - 2	94 (0.7%)	182 (0.8%)	p<0.001
	3 - 4	1,016 (7.1%)	1,378 (6.2%)	
	5 - 6	3,764 (26.3%)	5,254 (23.5%)	
	7 - 8	7,515 (52.5%)	11,769 (52.5%)	
	9 - 10	1,918 (13.4%)	3,821 (17.1%)	
Employment	Healthcare	2,495 (17.4%)	3,852 (17.2%)	p<0.001
	Education	1,639 (11.5%)	2,640 (11.8%)	
	Scientific and technical services	1,188 (8.3%)	2,564 (11.4%)	
	Finance and insurance	906 (6.3%)	1,072 (4.8%)	
	Arts, entertainment, and recreation	391 (2.7%)	863 (3.9%)	
	Retail	298 (2.1%)	353 (1.6%)	
	Manufacturing	305 (2.1%)	318 (1.4%)	
	Hospitality and food services	184 (1.3%)	370 (1.7%)	
	Other or not stated	6,901 (48.2%)	10,372 (46.3%)	

[1] Counties were stratified according to average weekly policy comprehensiveness below or above the median across counties for public health (4.83) for the period between April 22, 2020 – Dec 31, 2021. See Methods.
[2] Chi-squared test
[3] MacArthur-scale Subjective Social Status values between 1-10, combined into five groups.

Table S7. Characteristics of COVID-19 Citizen Science participants living in counties with low versus high average economic support policy scores.

Characteristic	Value	Low economic support policy activity (2.20 - 3.92) n = 13,518	High economic support policy activity (> 3.92 - 5) n = 23,193	p-value[2]
Age group (years)	18-34	1,817 (13.4%)	2,721 (11.7%)	p<0.001
	35-49	3,986 (29.5%)	6,470 (27.9%)	
	50-64	4,023 (29.8%)	7,271 (31.3%)	
	≥ 65	3,692 (27.3%)	6,731 (29.0%)	
Gender	Female	8,956 (66.3%)	15,179 (65.4%)	p=0.223
	Male	4,382 (32.4%)	7,676 (33.1%)	
	Other or not stated	180 (1.3%)	338 (1.5%)	
Race/ethnicity	Non-Hispanic White	11,309 (83.7%)	17,886 (77.1%)	p<0.001
	Hispanic, any race	889 (6.6%)	1,967 (8.5%)	
	Non-Hispanic Asian	535 (4.0%)	1,724 (7.4%)	
	Non-Hispanic Black	311 (2.3%)	469 (2.0%)	
	Non-Hispanic Other	474 (3.5%)	1,147 (4.9%)	
Subjective social status	1 - 2	97 (0.7%)	179 (0.8%)	p<0.001
	3 - 4	997 (7.4%)	1,397 (6.0%)	
	5 - 6	3,666 (27.1%)	5,352 (23.1%)	
	7 - 8	6,973 (51.6%)	12,311 (53.1%)	
	9 - 10	1,785 (13.2%)	3,954 (17.0%)	
Employment	Healthcare	2,460 (18.2%)	3,887 (16.8%)	p<0.001
	Education	1,586 (11.7%)	2,693 (11.6%)	
	Scientific and technical services	1,290 (9.5%)	2,462 (10.6%)	
	Finance and insurance	808 (6.0%)	1,170 (5.0%)	
	Arts, entertainment, and recreation	308 (2.3%)	946 (4.1%)	
	Retail	293 (2.2%)	358 (1.5%)	
	Manufacturing	301 (2.2%)	322 (1.4%)	
	Hospitality and food services	200 (1.5%)	354 (1.5%)	
	Other or not stated	6,272 (46.4%)	11,001 (47.4%)	

[1] Counties were stratified according to average weekly policy comprehensiveness below or above the median across counties for economic support (3.92) for the period between April 22, 2020 – Dec 31, 2021. See Methods.

[2] Chi-squared test

[3] MacArthur-scale Subjective Social Status values between 1-10, combined into five groups.

Table S8. Associations with self-reported anxiety[1].

covariate		estimate	lower CI	upper CI	point estimate p-value	omnibus p-value
Containment & closure policy[2]		-0.03	-0.20	0.15	0.77	
Public health policy[2]		-0.13	-0.38	0.12	0.31	
Economic support policy[2]		-0.12	-0.30	0.05	0.17	
Subjective Social Status[3]	SSS (numeric)	-1.12	-1.26	-0.97	< 0.0001	
	SSS^2 (quadratic)	0.05	0.04	0.06	< 0.0001	
Age[3]	Age (numeric)	-0.09	-0.11	-0.07	< 0.0001	
	Age^2 (quadratic)	5.8E-05	-1.2E-04	2.3E-04	0.51	
Gender[4]	Female	reference	--	--		< 0.0001
	Male	-0.94	-1.03	-0.85	< 0.0001	
	Other or not stated	1.46	1.11	1.80	< 0.0001	
Race/ethnicity[4]	Non-Hispanic White	reference	--	--	--	< 0.0001
	Hispanic, any race	0.08	-0.07	0.24	0.30	
	Non-Hispanic Asian	-0.85	-1.02	-0.68	< 0.0001	
	Non-Hispanic Black	-1.02	-1.31	-0.73	< 0.0001	
	Non-Hispanic Other	0.02	-0.17	0.22	0.81	
Employment[4]	Healthcare	reference	--	--	--	< 0.0001
	Education	0.40	0.25	0.55	< 0.0001	
	Scientific and technical services	0.14	-0.02	0.31	0.08	
	Finance and insurance	0.13	-0.07	0.33	0.21	
	Arts, entertainment, and recreation	0.79	0.55	1.03	< 0.0001	
	Retail	0.49	0.17	0.81	0.003	
	Manufacturing	-0.32	-0.65	0.01	0.06	
	Hospitality and food services	0.19	-0.15	0.53	0.28	
	Other or not stated	0.24	0.13	0.36	< 0.0001	
Average COVID-19 cases/100k in participants' county[5]		2.1E-03	1.5E-03	2.8E-03	< 0.0001	
Average COVID-19 deaths/100k in participants' county[5]		0.02	-0.04	0.07	0.52	

[1] Estimates, 95% confidence intervals (CI), and p-values from multivariable regression models for adjusted associations between GAD-7 anxiety scores and composite county-level COVID-19 policy indices, as those shown in Table 3 of the main manuscript, along with estimates for additional model covariates. Models were adjusted to account for random participant-level effects (using a random intercept for each participant), sociodemographic characteristics as fixed time-invariant factors, county-specific effects, calendar time (spline), and time-varying weekly COVID-19 case and death rates. Estimates for county-specific effects and calendar time (spline) are omitted from this table. See Methods.
[2] Mean county-level policy activity scores for the 4-week period prior to anxiety surveys, normalized to the maximum possible index value.
[3] Associations for Subjective Social Status (SSS) and age (in years at the time of enrollment) were estimated using numeric plus quadratic terms to account for nonlinear relationships.
[4] Point estimates for categorical variables indicate contrasts relative to the indicated arbitrary reference subgroup.
[5] Rolling weekly averages of county-level COVID-19 cases or deaths per 100,000 from the New York Times

Table S9. Adjusted associations between COVID-19 policy and anxiety using two-week, four-week, or eight-week rolling averages for county-level policy comprehensiveness[1].

Policy Type[2]	Preceding time period over which policy score is averaged		
	2 weeks	4 weeks	8 weeks
Containment and closure[3]	-0.01 (-0.18, 0.15)	-0.03 (-0.20, 0.15)	-0.03 (-0.21, 0.16)
Public health[4]	-0.03 (-0.27, 0.20)	-0.13 (-0.38, 0.12)	-0.29 (-0.55, -0.02)
Economic support[5]	-0.11 (-0.28, 0.07)	-0.12 (-0.30, 0.05)	-0.24 (-0.42, -0.05)

[1] Estimates and 95% confidence intervals from multivariable regression models for adjusted associations between GAD-7 anxiety scores and composite county-level COVID-19 policy indices. All models were adjusted to account for random participant-level effects (using a random intercept for each participant), sociodemographic characteristics as fixed time-invariant factors, county-specific effects, calendar time (spline), and time-varying weekly COVID-19 case and death rates. See Methods.
[2] Mean county-level policy activity scores for the 2-week, 4-week or 8-week period prior to anxiety surveys, normalized to the maximum possible index value. Estimates for 4-week policy averages are the same as those shown in Table 3 of the main manuscript (see Methods).
[3] Composite index of 13 containment and closure policy subcategories
[4] Composite index of 8 public health policy subcategories
[5] Composite index of 5 economic support subcategories

Table S10. Differential associations between COVID-19 policies and self-reported anxiety among population subgroups.

policy interaction[1]	characteristic	subgroup	estimate	lower CI	upper CI	p-value	omnibus p-value
containment & closure policy	Subjective Social Status	1 - 2	0.21	-0.67	1.08	0.64	0.0059
		3 - 4	0.12	-0.20	0.44	0.46	
		5 - 6	-0.09	-0.30	0.13	0.42	
		7 - 8	0.06	-0.13	0.24	0.56	
		9 - 10	-0.27	-0.49	-0.04	0.02	
	Age Group	18-34	0.09	-0.19	0.37	0.51	< 0.0001
		35-49	-0.03	-0.24	0.18	0.79	
		50-64	0.17	-0.03	0.37	0.10	
		≥ 65	-0.23	-0.43	-0.03	0.02	
	Gender	Female	0.05	-0.14	0.23	0.63	0.016
		Male	-0.17	-0.37	0.03	0.11	
		Other or not stated	0.12	-0.48	0.71	0.70	
	Race/ethnicity	Non-Hispanic White	-0.04	-0.22	0.14	0.64	0.61
		Hispanic, any race	0.05	-0.26	0.35	0.75	
		Non-Hispanic Asian	0.15	-0.18	0.48	0.37	
		Non-Hispanic Black	0.25	-0.42	0.92	0.47	
		Non-Hispanic Other	0.01	-0.36	0.39	0.94	
	Employment	Healthcare	-0.43	-0.66	-0.20	2.7E-04	< 0.0001
		Education	0.12	-0.14	0.37	0.37	
		Scientific and technical services	0.15	-0.11	0.41	0.26	
		Finance and insurance	-0.28	-0.63	0.06	0.11	
		Arts, entertainment, and recreation	0.56	0.15	0.97	0.01	
		Retail	-0.35	-0.94	0.25	0.26	
		Manufacturing	-0.26	-0.84	0.33	0.39	
		Hospitality and food services	1.05	0.45	1.64	5.8E-04	
		Other or not stated	0.00	-0.19	0.19	0.97	
public health policy	Subjective Social Status	1 - 2	-0.48	-2.18	1.23	0.59	0.10
		3 - 4	0.17	-0.38	0.72	0.55	
		5 - 6	0.09	-0.24	0.41	0.60	
		7 - 8	-0.17	-0.44	0.10	0.22	
		9 - 10	-0.44	-0.82	-0.06	0.02	
	Age Group	18-34	-0.26	-0.71	0.19	0.25	0.48
		35-49	-0.22	-0.53	0.09	0.16	
		50-64	-0.15	-0.45	0.14	0.32	
		≥ 65	0.03	-0.30	0.35	0.87	
	Gender	Female	-0.40	-0.67	-0.13	0.004	< 0.0001
		Male	0.42	0.09	0.75	0.01	
		Other or not stated	-0.12	-1.42	1.18	0.86	
	Race/ethnicity	Non-Hispanic White	-0.19	-0.45	0.06	0.14	0.0016
		Hispanic, any race	-0.63	-1.26	-0.006	0.05	
		Non-Hispanic Asian	0.74	0.05	1.43	0.04	
		Non-Hispanic Black	1.71	0.26	3.16	0.02	
		Non-Hispanic Other	0.27	-0.50	1.03	0.49	
	Employment	Healthcare	-0.02	-0.43	0.39	0.92	0.7
		Education	-0.36	-0.84	0.12	0.14	
		Scientific and technical services	0.04	-0.45	0.54	0.87	
		Finance and insurance	-0.55	-1.27	0.16	0.13	
		Arts, entertainment, and recreation	-0.53	-1.42	0.37	0.25	
		Retail	0.00	-1.25	1.25	1.00	
		Manufacturing	-0.01	-1.24	1.21	0.98	
		Hospitality and food services	0.36	-0.91	1.63	0.58	
		Other or not stated	-0.02	-0.32	0.28	0.89	

policy interaction[1]	characteristic	subgroup	estimate	lower CI	upper CI	p-value	omnibus p-value
economic support policy	Subjective Social Status	1 - 2	0.27	-1.28	1.83	0.73	0.51
		3 - 4	-0.17	-0.66	0.32	0.50	
		5 - 6	-0.26	-0.53	0.00	0.05	
		7 - 8	-0.12	-0.32	0.09	0.26	
		9 - 10	0.07	-0.25	0.39	0.67	
	Age Group	18-34	-0.36	-0.75	0.02	0.06	0.37
		35-49	-0.03	-0.27	0.21	0.81	
		50-64	-0.16	-0.40	0.07	0.17	
		≥ 65	-0.19	-0.47	0.09	0.17	
	Gender	Female	-0.19	-0.39	0.02	0.07	0.46
		Male	0.00	-0.28	0.28	0.99	
		Other or not stated	-0.31	-1.52	0.89	0.61	
	Race/ethnicity	Non-Hispanic White	-0.10	-0.29	0.09	0.30	0.27
		Hispanic, any race	-0.01	-0.62	0.59	0.96	
		Non-Hispanic Asian	-0.67	-1.42	0.08	0.08	
		Non-Hispanic Black	0.55	-0.51	1.61	0.31	
		Non-Hispanic Other	-0.58	-1.35	0.20	0.15	
	Employment	Healthcare	0.03	-0.31	0.38	0.86	0.16
		Education	-0.21	-0.62	0.21	0.33	
		Scientific and technical services	-0.13	-0.56	0.30	0.55	
		Finance and insurance	0.21	-0.37	0.80	0.48	
		Arts, entertainment, and recreation	0.73	-0.11	1.58	0.09	
		Retail	0.15	-0.89	1.18	0.78	
		Manufacturing	-0.33	-1.34	0.69	0.53	
		Hospitality and food services	-1.31	-2.44	-0.18	0.02	
		Other or not stated	-0.23	-0.47	0.02	0.07	

[1] Estimates, 95% confidence intervals and p-values from regression models that included interactions between composite policy scores in the participant's county of residence for the 4-week period prior to the anxiety survey response and selected participant characteristics. Each interaction model included interactions between all three composite policy index scores and a single participant characteristic (interactions were analyzed separately for Subjective Social Status, age group, gender, race/ethnicity, or employment). Each model was adjusted to account for random participant-level effects (using a random intercept for each participant), sociodemographic characteristics as fixed time-invariant factors, county-specific effects, calendar time (spline), and time-varying weekly COVID-19 case and death rates. See Methods.
